# Multiplex detection of eight different viral enteropathogens in clinical samples, combining RT-PCR technology with melting curve analysis

**DOI:** 10.1186/s12985-022-01789-z

**Published:** 2022-04-07

**Authors:** Wei Li, Weiwei Li, Lin Li, Yajun Guo, Jie Chen, Shiqiang Shang, Jianhua Mao

**Affiliations:** 1 Department of Clinical Laboratory, The Children’s Hospital, Zhejiang University School of Medicine, National Clinical Research Center for Child Health, Hangzhou, 310052 People’s Republic of China; 2 Jiangsu Bioperfectus Biotechnology Technologies Co, LTD, Taizhou, People’s Republic of China; 3 Department of Gastroenterology, The Children’s Hospital, Zhejiang University School of Medicine, National Clinical Research Center for Child Health, Hangzhou, 310052 People’s Republic of China; 4 Department of Nephrology, The Children’s Hospital, Zhejiang University School of Medicine, National Clinical Research Center for Child Health, 3333 Binsheng road, Hangzhou, 310052 People’s Republic of China

**Keywords:** Real time RT-PCR combined with melting curve, Virus, Acute diarrhea, Children

## Abstract

**Background:**

Early and accurate identification of infection viruses among children can benefit the personalized medical treatment and management, and reduce the future occurrence of serious symptoms. Thus, it is critical to develop a high-throughput multiplex real-time RT-PCR method to improve the accuracy and efficiency in routine clinical lab tests.

**Methods:**

We developed a real time RT-PCR combined with melting curve analysis (RRCMC) method for simultaneous detection of rotavirus A, B, C, norovirus GI and GII, adenovirus, astrovirus and sapovirus.

**Results:**

Stool samples were collected from 160 children with acute diarrhea and tested by RRCMC assay. A total of 71 patients were tested positive with norovirus, adenovirus or rotavirus. The RRCMC assay has high specificity. There is no internal cross-reaction among the 8 diarrhea viruses and no cross-reaction of other commonly intestinal pathogens and human genome. The limit detection was ranged from 1 × 10^2^ to 1 × 10^5^ nucleic acid copies/ml for each diarrhea virus.

**Conclusion:**

The RRCMC method is a suitable rapid clinical test for infectious viruses, with the advantages of high-throughput, low cost, high sensitivity and specificity.

## Background

Virus infection is one of the most common causes of acute diarrhea in children under 5 years of age. Pathogens of viral diarrhea include rotavirus, norovirus, adenovirus, astrovirus and sapovirus [1]. The main clinical manifestations of viral diarrhea covered diarrhea, vomit, and fever. Furthermore, acute diarrhea can also cause severe dehydration, leading to further complications and hospitalization [2]. Diarrheal diseases result in a large number of childhood deaths, the rotaviruses account for two fifth [3]. Rotaviruses are double-stranded RNA viruses forming a genus of the *Reoviridae* family and further subclassified into species A, B, C, and others. Rotavirus A is the primary known cause of severe gastroenteritis in infants and young children worldwide [4–6]. Norovirus is a non-enveloped, small RNA virus that contains a single stranded, positive-sense, polyadenylated RNA genome and it was associated with approximately one-fifth of all diarrhea cases [7]. Norovirus can be divided into five groups, of which norovirus GI and norovirus GII groups are mainly related to infections in humans [8]. Human adenovirus is non-enveloped virus which belongs to the family of *Adenoviridae* and has a linear 36-kb dsDNA genome. Human adenovirus is also recognized as an important cause of diarrhea in children [9]. Human astroviruses are small, non-enveloped positive-sense single-stranded RNA viruses in the *Astroviridae* family which are a major cause of diarrhea in children, the elderly, and immunocompromised people [10, 11]. Sapovirus is a member of the *Caliciviridae* family, and a single-stranded positive sense RNA virus. Sapovirus infections primarily affect children aged less than 5 years, causing mild to moderate diarrhea and outbreaks in all age groups [12].

In China, the antigen assay is the main tool for rapid screening of some viruses in many hospitals. However, such a technique is insensitive and cross-reactive [13, 14]. Serological tests are also widely used as diagnostic techniques in clinical practice. However, due to the time window for antibody to be produced, its usefulness for early diagnosis is small [6]. Moreover, both antigen and antibody detection are not applicable for multiple diarrhea viruses. Nucleic acid detection is widely used because of its high sensitivity, specificity and early diagnosis [8]. At present, clinical nucleic acid diagnosis is mostly based on the detection of DNA / RNA of diarrhea related virus. However, due to the limitation of the number of fluorescent dyes and labeled probes, it is impossible to detect more pathogens in one tube of detection solution. There are multiple PCR tests developed in previous studies with multiple detection function. However, these methods are either not real-time detection or not high-throughput, which is a challenge for laboratories that require high efficiency and productivity [15, 16]. Bennett et al. developed one-step multiplex real-time RT-PCR assay simultaneously detecting adenovirus, astrovirus, rotavirus and sapovirus in one tube which has reached the limit detection of fluorescent quantitative PCR in one system [17]. In this study, we developed a real time RT-PCR combined with melting curve (RRCMC) analysis for detecting rotavirus A, B, C, norovirus GI and GII, adenovirus, astrovirus and sapovirus in one PCR tube.

## Methods

### Study population

From March 2019 to May 2019, patients who met the following criteria were recruited in the present study: (1) children under 14 years of age, (2) Patients who visit Children’s Hospital of Zhejiang University School of Medicine in the inpatient wards and outpatient departments, (3) Primary diagnosis of acute diarrhea with suspected virus infections. Diarrhea was defined as three or more stools per day of unusual stool shape (liquid, watery, mucous or bloody purulent) within the previous 24 h. This study was approved by the medical ethics committee of the Children’s Hospital, Zhejiang University School of Medicine (No. 2019-IRB-082).

### Viruses DNA/RNA extraction

Stools were collected, and approximately 250 μl to 1 ml normal saline was added. The mixtures were centrifuged at 4000*g* at 20 °C for 30 s. A total of 200 μl supernatant was separated and DNA/RNA was extracted by SSNP-2000A nucleic acid automatic extraction instrument and Nucleic acid extraction or purification kits (Art. No: SDK60105). The above mentioned instrument and kit were from Jiangsu Bioperfectus Biotechnology Technologies Co, (Jiangsu, China).

### Real time RT-PCR combined with melting curve analysis

The principle of the RRCMC test was reported in a previous study [18] as follows: In each fluorescence channel, four Taqman probes labeled with the same fluorescence dyes and eight primers was designed to target four viruses (The length of each probe is inconsistent), Rotavirus C, Astrovirus, Rotavirus B, and Norovirus GI were detected by a FAM probe and Rotavirus A, Sapovirus, Norovirus GII, and Adenovirus were detected by a VIC probe. The each probe is designed with a fully complementary 3′ phosphorylation closed melting curve oligonucleotide. Taqman probes with sequences of varying length can be distinguished if the probes themselves, independent of hybridising to 3′ phosphorylation closed melting curve oligonucleotide, have different melting temperatures. If a virus target is present, its corresponding probe is consumed during PCR amplification, no probe can complementary pairing with 3′ phosphorylation closed melting curve oligonucleotide and the corresponding melting curve is reduced or has disappeared. The negative control has all melting curves. Comparing melting profiles of the probes with negative control after the reaction reveals which probes have been consumed and melting curve decreased; this in turn indicates which targets are present in a sample. The primers and probes were shown in Table 1.

**Table 1:** Sequences of probes and primers

Virus	Primer/probe	Sequence (5′–3′)	Length of amplicons (bp)
Rotavirus A	F	ATTGAAGCAGAWTMTGATTCTG	147
	R	ACAAATCYTYRATCAATTGCAT	
	P	VIC-ATTCAGATAGTGATGAYGGWAAATGTA-BHQ1	
	Ph	TACATTTCCCTTCATCACTATTTGAAT-Phosphorylation	
Rotavirus B	F	CTCAGTTCTTGTAGTATATTAGCTATCG	133
	R	CTCCACGAGGCAATTTGATA	
	P	FAM-AACGCTTCTATGGATTTTAATGTTTTTCTTCAG-BHQ1	
	Ph	CTGAATAAAAACTTTAATATCCATATAAGTGTT-Phosphorylation	
Rotavirus C	F	CCATTCTCTTCATTCTTTTCATTT	215
	R	TGGAAAATAAATACATAAAGATTCAAC	
	P	FAM-TAATTGTGTAGAGTGGTCACAAGGTCAGATG-BHQ1	
	Ph	CATCTGATCTTGTTACCACTTTACATAATTA-Phosphorylation	
Norovirus GI	F	TTCCATGATTTGAGTTTGTGG	100
	R	GGTGCCATCCATGTTTGTT	
	P	FAM-CAGGAGACCGCGATCTCCTGC-BHQ1	
	Ph	GCATGAGATCTCGTTTTCCTG-Phosphorylation	
Norovirus GII	F	AAAGACTGTCTGAAGCTACTCCT	105
	R	GACCAGATTTAGTGATAAACACAAT	
	P	VIC-CTCCCCTGCAAGAGGCCATTT-BHQ1	
	Ph	AAATGTCCTTTTGTAGGGTAG-Phosphorylation	
Astrovirus	F	AATTGCTCAAAGAGGAAATAGA	199
	R	GCAAGTATVCCATTGATTTCAT	
	P	FAM-AGAACTGCAATGGAACGTGAGATGAAG-BHQ1	
	Ph	CTTCTTCTCATGTTCTATTGTAGTTCT-Phosphorylation	
Sapovirus	F	CCAATGTCAATTACGACCAG	115
	R	TCCATTTCAAACACTAATTTGG	
	P	VIC-CCACCTACGAATCTTGGTTCATAGGCG-BHQ1	
	Ph	CGCTTATGAATCAAGATTCGTATGTGG-Phosphorylation	
Adenovirus	F	CTTACAAAGTGCGCTTTACG	110
	R	AGGGTTTAAAGCTGGGGC	
	P	VIC-CATGGCCAGCACCTACTTTGACATC-BHQ1	
	Ph	GATTTCAAATTAGTTGCTGTCCTTG-Phosphorylation	

The probe (P) is fully complementary 3′ phosphorylation closed melting curve oligonucleotide (Ph)

*F* Forward primer; *R* Reverse primer; *P* probe; *Ph* 3′ phosphorylation closed melting curve oligonucleotide

The reagents were supported by Jiangsu Bioperfectus Biotechnology Technologies Co, China. PCR reactions system in a final volume of 30 μl consisted of 4 components: 10 μl of nucleic acid amplification reaction solution (dNTPs、Tris、KCl、MgCl_2_), 10 μl primer/probe mix (primer: 200–300 nM/μL, Taqman probe: 100–150 nM/μL, 3′ phosphorylation closed melting curve oligonucleotide: 100–300 nM/μL), 5 μl Enzyme mixture (M-MLV and *Taq*), and 5 μl template. Amplification reactions and melting profiles were performed in a real-time PCR SLAN-96P system (HONGSHI, China). The thermal profile was: 50 °C for 20 min; 95 °C for 5 min; 8 cycles of 95 °C for 10 s and 60 °C for 40 s; 40 cycles of 95 °C for 10 s, 55 °C for 30 s, and 58 °C for 15 s. Fluorescence measurements were recorded during the read steps at 58 °C. Post-amplification melting profiling was carried out under the following conditions: after the last cycle of PCR, heat at 95 °C for 15 s, cool to 20 °C and slowly increase the temperature at a speed of 0.03 °C/s. The fluorescence emission data is continually collected during the rising temperatures. The negative derivative of the emission reading, with respect to temperature, is plotted against the temperature to form melting curves, and the peak of the curve corresponds to the Tm of the probe. If a FAM probe positive, according to negative control melting curve, the temperature position of melting curve is reduced and can be used to confirm which virus is infected: rotavirus C (29 ± 1 °C), astrovirus (39 ± 1 °C), astrovirus (48 ± 1 °C), astrovirus (58 ± 1 °C). If a VIC probe positive, according to negative control, the temperature position of rotavirus A is 29 ± 1 °C, sapovirus is 40 ± 1 °C, norovirus GII is 49 ± 1 °C and adenovirus is 57 ± 1 °C.

## Results

### Character of RRCMC assay

**Fig. 1:**
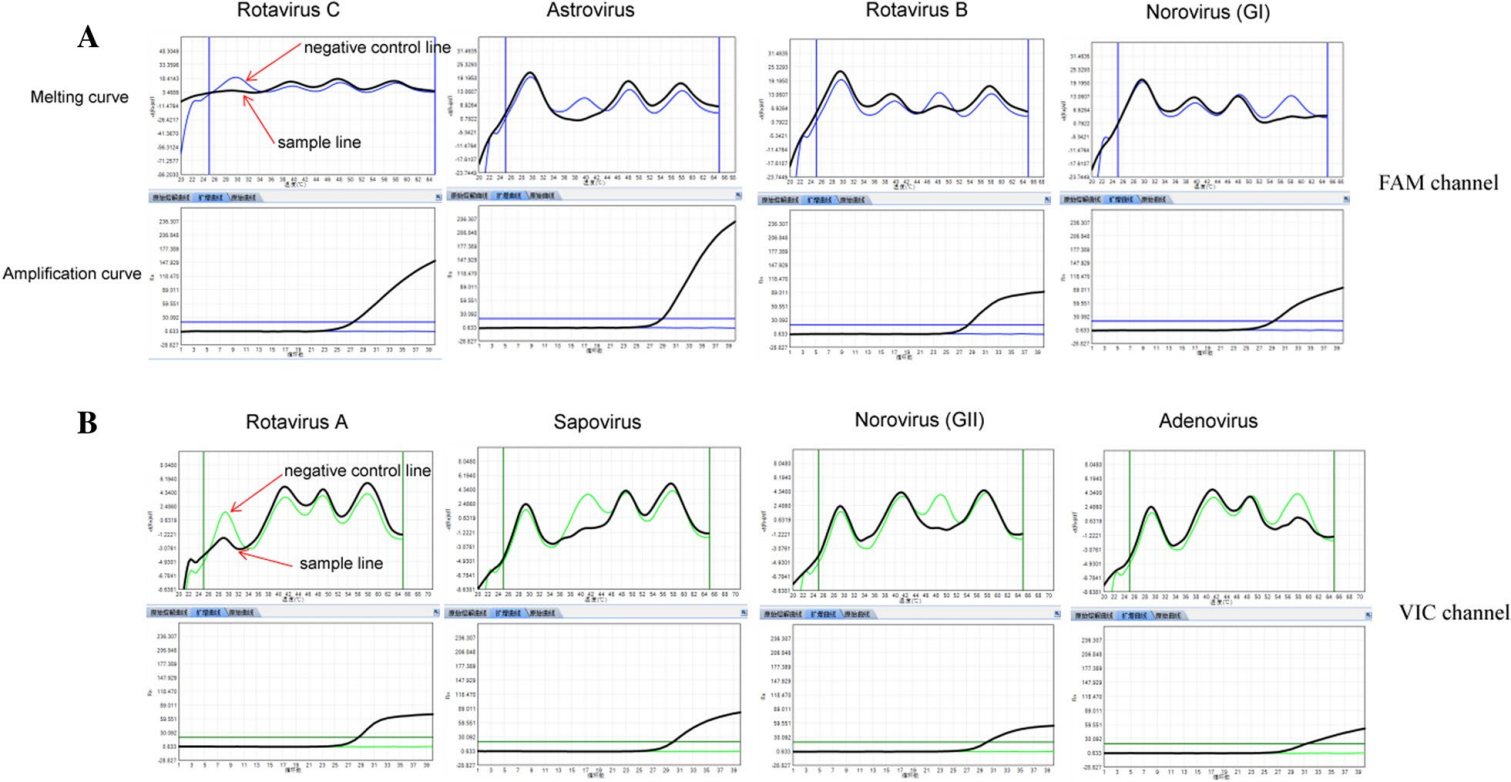
Amplification and melting curve of RRCMC assay. **A** Rotavirus C, astrovirus, rotavirus B, and norovirus GI were detected by FAM channel, and each virus was confirmed by temperature position of melting curve (Blue melting curve is the negative control and black melting curve is the sample). **B** Rotavirus A, sapovirus, norovirus GII, and adenovirus were detected by VIC channel, and each virus was confirmed by temperature position of melting curve (Green melting curve is the negative control and black melting curve is the sample). Amplication curve: ordinates is fluorescence value and abscissae is number of cycles; Melting curve: ordinates is Fluorescence value and abscissae is temperature

Clinical strains of rotavirus A, rotavirus B, rotavirus C, GI, norovirus GII adenovirus, sapovirus, and astrovirus were tested by RRCMC assay. As shown in Fig. 1, comparing to negative control, and according to the melting curve temperature, the order of detection for virus was rotavirus C, astrovirus, rotavirus B, and norovirus GI in FAM channel. In VIC channel of RRCMC assay, the order of detection for virus were rotavirus A, sapovirus, norovirus GII, and adenovirus with melting curve temperature rising.

### Sensitivity and specificity

In order to confirm sensitivity of RRCMC assay, the target fragment of each virus was cloned into pGH plasmid vector. And for this, we prepared serial tenfold dilutions from 10^7^ copies/mL to 10^2^ copies/mL. The results showed that the detection limitation of the RRCMC method was at least as follows: rotavirus A: 1.0 × 10^2^, rotavirus B: 2.0 × 10^3^, rotavirus C: 1.3 × 10^4^, adenovirus: 1.0 × 10^4^, GI: 1.0 × 10^5^, GII: 1.0 × 10^5^, astrovirus: 4.0 × 10^3^, sapovirus: 4.0 × 10^3^. To determine the specificity of RRCMC assay, clinical strains of rotavirus A, rotavirus B, rotavirus C, GI, GII adenovirus, sapovirus, and astrovirus were used as controls and all clinical strains were confirmed by commercial real-time PCR assay (Jiangsu Bioperfectus Biotechnology Technologies Co, LTD). All clinical strains were collected from stool specimen and stored at − 80 °C until used. For RRCMC assay, all were detected positive and there were no false positive or false negative results. There is no internal cross-reaction between 8 viruses by using RRCMC assay while each virus was positive. No fluorescence was detected and no cross-reaction was found in DNAs extracted from the human genome, *Salmonella*, *Shigella*, diarrhea causing *Escherichia coli*, *Yersinia enterocolitica*, *Campylobacter jejuni*, *Aeromonas hydrophila*, *Vibrio parahaemolyticus* in this test. All clinical bacterial strains were confirmed by bacterial culture and stored at − 20 °C until used.

### Results of clinical samples

**Fig. 2:**
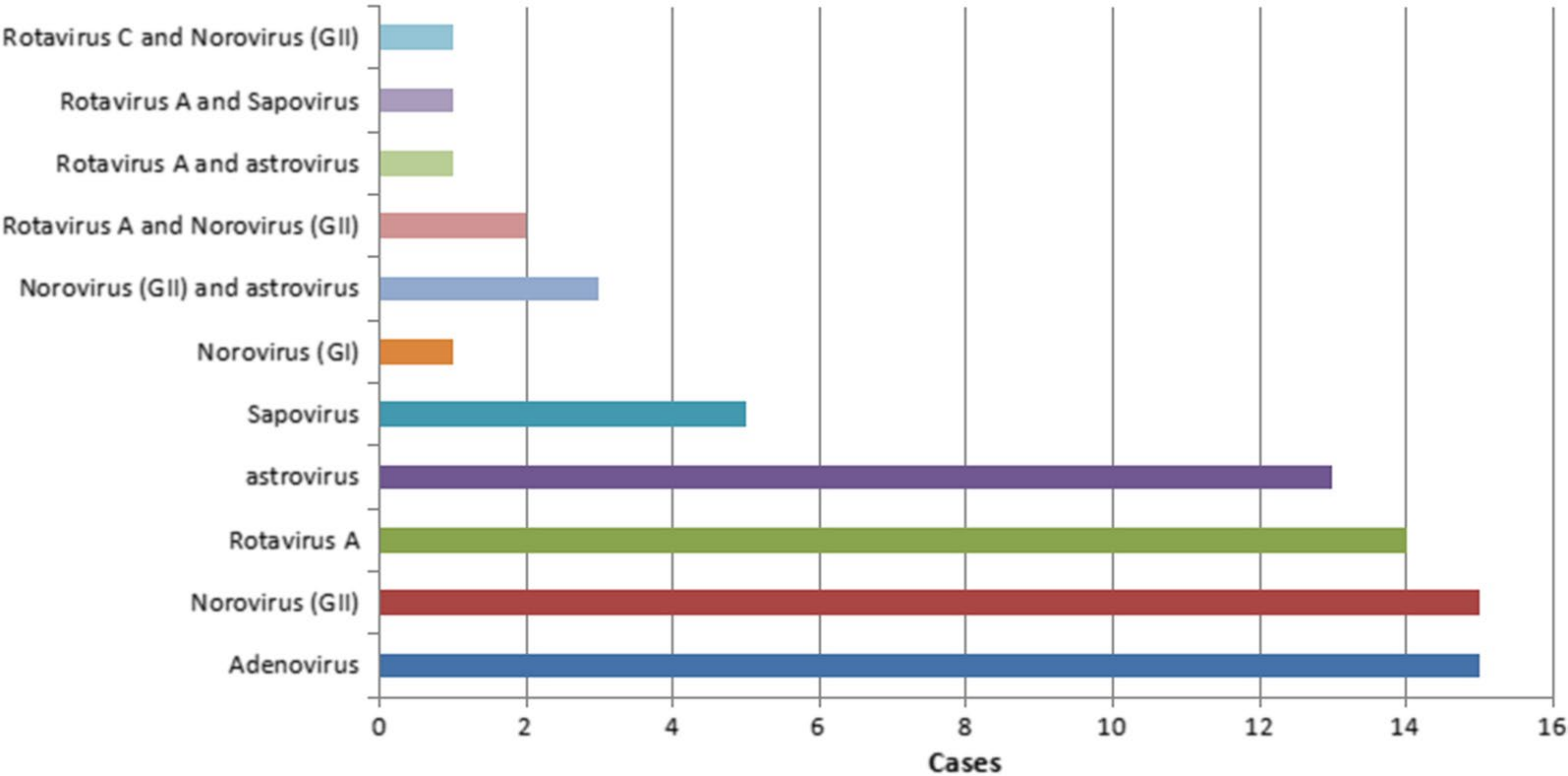
Distribution of various viruses in 71/160 patients with acute diarrhea using the RRCMC assay

A total of 160 cases clinically diagnosed as acute diarrhea were enrolled into this study from March 2019 to May 2019, and stool samples were collected and tested by RRCMC assay. There were 108 samples from boys while 52 samples from girls, which yielded a male-to-female ratio of 2.08:1. A total of 71 samples were tested positive by RRCMC assay with positive rate of 44.38%. Of these, 48 (44.44%, 48/108) were from boys and 23 (44.23%, 23/52) were from girls. As shown in Fig. 2, among 71 positive samples, norovirus has the highest positive rate of 10.00% (16/160) comprising 15 cases of norovirus GII (9.38%, 15/160) and 1 case of GII (0.63%, 1/160), successively followed by adenovirus (9.38%, 15/160), rotavirus A (8.75%, 14/160) and astrovirus (8.13%, 13/160), sapovirus (3.13%, 5/160), norovirus (GII) and astrovirus co-infection (1.88%, 3/160), rotavirus A and norovirus (GII) co-infection (1.25%, 2/160), rotavirus A and astrovirus co-infection (0.63%, 1/160), rotavirus A and sapovirus co-infection (0.63%, 1/160), rotavirus C and norovirus (GII) co-infection (0.63%, 1/160). We also analyzed the age distribution of virus positive children. The median age of children with rotavirus A infection was 1.33 years old (0.53–7.90), norovirus (GII) was 1.25 years old (0.15–13.58), adenovirus was 1.74 years old (0.50–11.74), astrovirus was 1.33 years old (0.15–8.82) and sapovirus was 2.58 years old (1.08–5.33).

## Discussion

Acute diarrhea is still a major problem for children especially in developing countries, and it is associated with a large number of morbidity, mortality and economical burden. [19]Accurate diagnosis of viral diarrhea can avoid the abuse of antibiotics after misdiagnosis as bacterial diarrhea, and targeted treatment can be initiated at an early stage as soon as possible. At the same time, the clinical symptoms of these viruses are similar in the early stage of infection, but there are great differences in the clinical severity in the later stage. Rotavirus A can cause severe clinical symptoms such as dehydration and electrolyte disturbance, while Enteroadenovirus can cause mild to moderate dehydration in most children [20]. Norovirus and sapovirus can cause statistically less clinical symptoms in children than rotavirus A [21, 22]. Our results also revealed that the median age of children infected with each diarrhea virus was very close (1–2 years), except for astrovirus. Therefore, the accurate identification of viral pathogens at early infectious period can individualize the treatment and management of children to avoid the occurrence of serious clinical symptoms. Many diagnostic techniques have been used for the detection of diarrhea viruses, including serological methods, immune electron microscopy, and enzyme-linked immunosorbent assays (ELISA). However, these techniques are time-consuming and are low in sensitivity and specificity [2]. In order to detect common viruses in children with diarrhea diseases, the high throughput, multiplex real-time RT-PCR was developed for the detection of adenovirus, astrovirus, rotavirus or sapovirus from stool samples in previous study [15–17]. However, the detection capacity of this method is not large enough.

This paper describes the development and validation of a real time RT-PCR combined with melting curve assay, which will allow rapid and simultaneous detection of rotavirus A, B, C, norovirus GI and GII, adenovirus, astrovirus and sapovirus in stool samples, it only takes about 3–5 h to finish the testing process. The cost of RRCMC assay is about 10 dollar per sample, thus it is a relatively inexpensive novel diagnostic method. Because of the conservative characteristics of primers and probes and the control of melting curve, RRCMC assay has high specificity. There is no internal cross-reaction among 8 diarrhea viruses. The specificity of the assay was also confirmed by testing a panel of other commonly intestinal pathogens and no-template controls and no false positive results were encountered. Previous study suggested that there was a significant correlation between severity and the virus copies, and high sensitivity of PCR assay is helpful for early diagnosis [16]. Our study showed that the detection limit of the RRCMC method was ranged from 1 × 10^2^ to 1 × 10^5^ copies/ml for each diarrhea associated virus. Among them, the sensitivity of rotavirus detection is the highest (1 × 10^2^ copies/ml) and the sensitivity of norovirus (GI and GII) detection is the lowest (1 × 10^5^ copies/ml). This may due to the instability of the whole system and the preference of primer amplification. In further study, we will modified the primers and reaction system to improve the detection sensitivity.

In 160 patients with acute diarrhea, a total of 71 patients were tested positive by RRCMC. In our study, rotavirus A, C, norovirus GI and II, adenovirus, astrovirus and sapovirus were detected by RRCMC, and norovirus, adenovirus and rotovirus were the most common viruses caused acute diarrhea disease in children in precious studies. Interestingly, RVC was only detected in one case, which was co-infected with norovirus GII and rotavirus B was not detected in this study. In further studies, we will enroll more clinical samples from children with acute diarrhea disease to evaluate the effect of RRCMC method and epidemiology of eight viruses.

## Conclusions

In this study, we developed a real time RT-PCR combined with melting curve technique for detecting rotavirus A, B, C, norovirus GI and GII, adenovirus, astrovirus and sapovirus in one PCR tube. This novel method has the advantages of low cost because of single tube reaction, high sensitivity and specificity, and is suitable for general fluorescent quantitative PCR instrument, which is suitable for rapid clinical application. So the RRCMC method would be a valuable supplementary test in clinical practice.

## Data Availability

All data generated or analysed during this study are included in this published article.

## Abbreviations

RT-PCR: Reverse transcription-polymerase chain reaction
RRCMC: Real time RT-PCR combined with melting curve
ELISA: Enzyme-linked immunosorbent assays

## References

[1] Ouyang Y, Ma H, Jin M, Wang X, Wang J, Xu L, Lin S, Shen Z, Chen Z, Qiu Z, Gao Z, Peng L, Li J (2012). Etiology and epidemiology of viral diarrhea in children under the age of five hospitalized in Tianjin. China Arch Virol.

[2] Clark B, McKendrick M (2004). A review of viral gastroenteritis. Curr Opin Infect Dis.

[3] Bányai K, Estes MK, Martella V, Parashar UD (2018). Viral gastroenteritis. Lancet.

[4] Soares-Weiser K, Bergman H, Henschke N, Pitan F, Cunliffe N (2019). Vaccines for preventing rotavirus diarrhoea: vaccines in use. Cochrane Database Syst Rev..

[5] Li W, Xiang W, Li C, Xu J, Zhou D, Shang S (2020). Molecular epidemiology of rotavirus A and adenovirus among children with acute diarrhea in Hangzhou. China Gut Pathog.

[6] Crawford SE, Ramani S, Tate JE, Parashar UD, Svensson L, Hagbom M, Franco MA, Greenberg HB, O'Ryan M, Kang G, Desselberger U, Estes MK (2017). Rotavirus infection. Nat Rev Dis Primers.

[7] Ahmed SM, Hall AJ, Robinson AE, Verhoef L, Premkumar P, Parashar UD, Koopmans M, Lopman BA (2014). Global prevalence of norovirus in cases of gastroenteritis: a systematic review and meta-analysis. Lancet Infect Dis.

[8] Chhabra P, de Graaf M, Parra GI, Chan MC, Green K, Martella V, Wang Q, White PA, Katayama K, Vennema H, Koopmans MPG, Vinjé J (2019). Updated classification of norovirus genogroups and genotypes. J Gen Virol.

[9] Vinjé J (2015). Advances in laboratory methods for detection and typing of norovirus. J Clin Microbiol.

[10] Gelaw A, Pietsch C, Liebert UG (2019). Genetic diversity of human adenovirus and human astrovirus in children with acute gastroenteritis in Northwest Ethiopia. Arch Virol.

[11] Johnson C, Hargest V, Cortez V, Meliopoulos VA, Schultz-Cherry S (2017). Astrovirus pathogenesis. Viruses.

[12] Sánchez GJ, Mayta H, Pajuelo MJ, Neira K, Xiaofang L, Cabrera L, Ballard SB, Crabtree JE, Kelleher D, Cama V, Bern C, Oshitani H, Gilman RH, Saito M, Sapovirus Working Group (2018). Epidemiology of sapovirus infections in a birth cohort in Peru. Clin Infect Dis..

[13] Parashar UD, Nelson EA, Kang G (2013). Diagnosis, management, and prevention of rotavirus gastroenteritis in children. BMJ..

[14] Xiang W, Peng Z, Xu J, Shen H, Li W (2020). Evaluation of a commercial latex agglutination test for detecting rotavirus A and human adenovirus in children's stool specimens. J Clin Lab Anal..

[15] Kulis-Horn RK, Tiemann C (2020). Evaluation of a laboratory-developed test for simultaneous detection of norovirus and rotavirus by real-time RT-PCR on the Panther Fusion® system. Eur J Clin Microbiol Infect Dis.

[16] De Grazia S, Bonura F, Pepe A, Li Muli S, Cappa V, Filizzolo C, Mangiaracina L, Urone N, Giammanco GM (2019). Performance evaluation of gastrointestinal viral ELIte panel multiplex RT-PCR assay for the diagnosis of rotavirus, adenovirus and astrovirus infection. J Virol Methods.

[17] Bennett S, Gunson RN (2017). The development of a multiplex real-time RT-PCR for the detection of adenovirus, astrovirus, rotavirus and sapovirus from stool samples. J Virol Methods.

[18] Fu G, Miles A, Alphey L (2012). Multiplex detection and SNP genotyping in a single fluorescence channel. PLoS ONE.

[19] Guarino A, Dupont C, Gorelov AV, Gottrand F, Lee JK, Lin Z, Lo Vecchio A, Nguyen TD, Salazar-Lindo E (2012). The management of acute diarrhea in children in developed and developing areas: from evidence base to clinical practice. Expert Opin Pharmacother.

[20] Glass RI, Parashar UD, Estes MK (2009). Norovirus gastroenteritis. N Engl J Med.

[21] Siqueira JAM, Oliveira DS, Carvalho TCN, Portal TM, Justino MCA, da Silva LD, Resque HR, Gabbay YB (2017). Astrovirus infection in hospitalized children: molecular, clinical and epidemiological features. J Clin Virol.

[22] Sakai Y, Nakata S, Honma S, Tatsumi M, Numata-Kinoshita K, Chiba S (2001). Clinical severity of Norwalk virus and Sapporo virus gastroenteritis in children in Hokkaido. Jpn Pediatr Infect Dis J.

